# *Staphylococcus epidermidis’* Overload During Suckling Impacts the Immune Development in Rats

**DOI:** 10.3389/fnut.2022.916690

**Published:** 2022-07-04

**Authors:** Carla Morales-Ferré, Àngels Franch, Margarida Castell, Mónica Olivares, María J. Rodríguez-Lagunas, Francisco J. Pérez-Cano

**Affiliations:** ^1^Physiology Section, Department of Biochemistry and Physiology, Faculty of Pharmacy and Food Science, University of Barcelona, Barcelona, Spain; ^2^Nutrition and Food Safety Research Institute (INSA-UB), Santa Coloma de Gramenet, Spain; ^3^Biosearch Life SA, Granada, Spain

**Keywords:** mastitis, *Staphylococcus epidermidis*, suckling rat, immune system, intestinal gene expression, immunoglobulins

## Abstract

Mastitis is an inflammation of the mammary gland occurring in 3–33% of the breastfeeding mothers. The majority of mastitis cases have an infectious etiology. More than 75% of infectious mastitis are caused by *Staphylococcus epidermidis* and *Staphylococcus aureus* and involves breast milk microbiota alteration, which, may have an impact in lactating infant. The aim of this study was to analyze in rats during the suckling period and later in life the impact of a high and a low overload of *Staphylococcus epidermidis*, similarly as it occurs during the clinical and the subclinical mastitis, respectively. From days 2 to 21 of life, suckling rats were daily supplemented with low (Ls group) or high (Hs group) dose of *S. epidermidis*. Body weight and fecal humidity were periodically recorded. On days 21 and 42 of life, morphometry, hematological variables, intestinal gene expression, immunoglobulin (Ig) and cytokine profile and spleen cells’ phenotype were measured. Although no differences were found in body weight, Ls and Hs groups showed higher body length and lower fecal humidity. Both doses induced small changes in lymphocytes subpopulations, reduced the plasma levels of Ig and delayed the Th1/Th2 balance causing a bias toward the Th2 response. No changes were found in cytokine concentration. The low dose affected the Tc cells intestinal homing pattern whereas the high dose had an impact on the hematological variables causing leukocytosis and lymphocytosis and also influenced the intestinal barrier maturation. In conclusion, both interventions with *Staphylococcus epidermidis* overload during suckling, affects the immune system development in short and long term.

## Introduction

Mastitis is an inflammation of the mammary gland that can be accompanied or not by infection, which, can be fatal unless properly treated (1).

The incidence of mastitis occurs in 3% up to 33% of the breastfeeding mothers (2). Although this disease can appear in any moment of the breastfeeding, the 74–95% of the cases are reported to occur during the first 12 weeks of breastfeeding (1). The risk factors that predispose women to suffer from mastitis are the use of antibiotics during pregnancy, delivery or breastfeeding, previous mastitis, cracked or sore nipples, stress, incorrect breastfeeding, autoimmune diseases, immune deficit, smoking, and age of the mother (younger than 21 years and older than 35 years have less incidence) (1, 3–5).

The majority of mastitis cases have an infectious etiology (5, 6). Infectious mastitis cause a perturbation in breast milk microbiota increasing the abundance and proportion of the causal bacteria and reducing drastically the rest of the bacterial groups (7). Although *Staphylococcus* and *Streptococcus* genera are normally present in mammary gland microbiota (7), they are the main cause of these infectious mastitis, specifically species of the *Staphylococcus* genus. Thus, more than 75% of mastitis are caused by *Staphylococcus epidermidis* (*S. epidermidis*) and *Staphylococcus aureus* (8). *S. epidermidis* is a catalase-positive, coagulase-negative, gram positive, and facultative anaerobe bacterium (9) that causes subacute mastitis, which is characterized by sharp, needling pain and a burning sensation in the breast (5, 6).

The antibiotic therapy during 7–14 days is the first-line treatment against infectious mastitis (2, 6). However, there are bacteria resistant to antibiotics and with the ability to form *biofilms*, facilitating the recurrency or the chronicity of mastitis (6). Moreover, to treat the symptomatology, antipyretics, analgesics and local anti-inflammatories are widely used (5).

*In vitro* models of mastitis are used for studying the interaction between the pathogen and particular host cells but do not allow the study of the complexity of the mammary gland, the impact on the breast milk and even less, the consequences of the process on the descendance. Therefore, the use of animal models is necessary. Rodents such as mice and rats are good alternative (10) due to their easier manipulation, lower cost and the higher number of animals per litter that allow the obtention of valid statistics in a short period of time (11, 12). There are many procedures to induce mastitis in mice or rats such as the use of pathogens and lipopolysaccharide (11, 13–16). However, the studies of mastitis are focused on the mother’s health, rather than on the consequences of the intake of the disbiotic milk by the pups in the short and in the long term. Moreover, the suckling rat is a good animal model to study the development of the immune system during early life because newborn rats have and immature immune system, that develops during breastfeeding and weaning and allows the study of their progress in few weeks (12).

Our hypothesis is that during both clinical and subclinical mastitis -induced by an overgrowth of *S. epidermidis*-, an overload of this bacteria is transferred *via* breast milk to the lactating babies which may have an impact in their growth and immune system development.

Hence, the aim of this study is to analyze the impact of a high and low overload of *S. epidermidis* in suckling rats during the suckling period, similarly as it occurs during the clinical and the subclinical mastitis, respectively. For assessing the influence of this particular bacteria overload during suckling two timepoints will be studied, the first one at the end of suckling period, and the second one 3 weeks after weaning. Growth and immunity variables will be evaluated in both periods of time to establish the influence of the overload in the immune development.

## Materials and Methods

### Animals

Pregnant Wistar rats (G14) provided by Janvier (Le Genest St Isle, France) were individually housed in cages (2184L Eurostandard Type II L, Tecniplast, West Chester, PA, United States), monitored daily and allowed to deliver at term. The cages contained tissue papers (Gomà-Camps S.A.U., La Riba, Spain) and bedding of large fibrous particles (Souralit 1035, Bobadeb S.L., Santo Domingo de la Calzada, Spain) as cage enrichment. The day of birth was registered as day 1 of life. The following day, litters were randomly assigned to three experimental groups and were unified to 8 pups per lactating dam with a similar proportion (40–60%) of each sex in each litter. Pups had free access to maternal milk and rat diet. Dams were fed with a commercial diet corresponding to the American Institute of Nutrition 93 G formulation (17) (Teklad Global Diet 2014, Envigo, Indianapolis, IN, United States) and water *ad libitum*. Animal handling was performed during the first hours of the light phase on a scheduled basis, to limit the disturbance and biological rhythms’ influence. After separating all the mothers and keeping the pups in the home-cage, handling and oral administration was performed once a day. Afterward, the dam was reunited with her litter. Animals were housed under controlled conditions of temperature (20–24°C) and humidity (40–60%) in a 12 h light–12 h dark cycle (lights on at 8:00 a.m and lights off at 8:00 p.m), at the Faculty of Pharmacy and Food Science animal facility (University of Barcelona, Spain). Cage cleaning was performed weekly. All experimental procedures were conducted in accordance with the institutional guidelines for the care and use of laboratory animals and were approved by the Ethical Committee for Animal Experimentation of the University of Barcelona and the Catalonia Government (CEEA/Ref. 308/19 and PAMN/Ref.10542, respectively), in full compliance with national legislation following the EU-Directive 2010/63/EU for the protection of animals used for scientific purposes. Sample size estimation was calculated by the Appraising Project Office’s program from the Universidad Miguel Hernández de Elche (Alicante, Spain). The minimal number of animals to provide statistically significant differences among groups, using plasma immunoglobulin (Ig) G as a variable and assuming that there was no dropout rate and type I error of 0.05 (two-sided), was three litters per group, as in previous studies (18, 19).

### Experimental Design and Sample Collection

Upon natural delivery, pups were distributed into three groups of 24 animals each (3 litters of 8 animals/group): the reference (REF) group and two groups supplemented with an overload of *S. epidermidis* CECT 9816 (isolated by Biosearch Life from breast milk in a patient with subclinical mastitis). One group received a low dose (Ls) and the other a high dose (Hs) which may reflect the bacterial load received by the pups during a subclinical and a clinical infectious mastitis, respectively. *S. epidermidis* strain was provided by Biosearch Life (Granada, Spain). Suckling rats were orally administered once daily during the whole suckling period (21 days), as previously described (20), the REF group was supplemented with the vehicle (50 μL of glycerol 15%) and the groups supplemented with *S. epidermidis* received each week the same volume of 50 μL with an increasing dose of the bacterium in glycerol 15%. The Ls group was supplemented with 4.0 × 10^8^ the first week of life, 9.0 × 10^8^ the second and 1.4 × 10^9^ colony forming units (CFU)/mL the third whereas the Hs group received 10 times the previous doses (4.0 × 10^9^ the first, 9.0 × 10^9^ the second and 1.4 × 10^10^ CFU/mL the third week of life).

The doses were proposed considering the relative abundance of the *Staphylococcus* in the breast milk of healthy mothers and from those suffering from mastitis (21) in relation to the proportion of these bacteria present in rat breast milk (22, 23) and the amount of milk received in each week of lactation (24).

Pup body weight was recorded daily. Moreover, the naso-anal and tail lengths were measured to determine the body/tail ratio. In addition, body mass index (BMI) was calculated as body *weight/lenght^2^* (g/cm^2^), and the Lee Index was calculated as *(weight^0.33^/length) × 1,000* (g^0.33^/cm).

During the study, fecal samples were obtained after gentle abdominal massage, to determine changes in the humidity. The sampling was always performed at the same time period, after the pups were separated from their mothers and weighted, and prior to the oral administration.

Animals were euthanized at two different time points by selecting 4 pups from each dam maintaining sex equality on each day of sacrifice: half of the litter on day 21 (the last day of *S. epidermidis* intervention) and the other half on day 42 (3 weeks after the intervention; the end of the study). Rats were intramuscularly anesthetized with ketamine (90 mg/kg) (Merial Laboratories S.A., Barcelona, Spain) and xylazine (10 mg/kg) (Bayer A.G., Leverkusen, Germany) and exsanguinated by cardiac puncture in heparin-treated tubes. First, blood was immediately used to count platelets and white and red blood cells and related variables (Spincell3 automated hematology analyzer, MonLab, Barcelona), following the manufacturer’s instructions. Plasma was obtained to determine the Ig and cytokine concentrations. The weights of the liver, stomach, spleen, thymus, small intestine, and large intestine were also recorded. Moreover, the lengths of the small and large intestines were also measured. The spleen and the small intestine were collected for further analysis. A portion of 1 cm of the central part of the small intestine was immediately conserved in RNAlater^®^ (Ambion, Applied Biosystems, Austin, TX, United States), incubated at 4°C overnight and stored at –20°C until PCR analysis whereas spleen was used for lymphocytes immunofluorescence staining.

### Fecal Humidity and pH of the Cecal Content

Once a week, fecal samples were obtained, weighted, and dried in a stove at 60°C during 24 h. Then, feces were weighted again to calculate the percentage of humidity. For pH determination, cecal samples from days 21 and 42 were used. The measurement was performed with a micropH 2001 pH meter (Crison Instruments, Barcelona, Spain) using a 5,207 pH electrode for surfaces.

### Quantification of Immunoglobulins and Cytokines

At the end of the *S. epidermidis* intervention (day 21) and 3 weeks later (day 42), the concentration of IgA in the gut wash was measured by a sandwich ELISA technique with the Rat IgA ELISA quantification test from Bethyl Laboratories (Montgomery, TX, United States), as previously described (25). Briefly, a 96-well plate (Nunc MaxiSorp, Wiesbaden, Germany) was coated with 2 μg/mL of the capture antibody. After blocking, the standard and the samples were incubated. Then, detection antibody was added and, after washing, an o-phenylenediamine dihydrochloride-H_2_O_2_ (Sigma-Aldrich) solution was added. Absorbance was measured in a microplate photometer (LabSystems Multiskan) and data were interpolated by Multiskan Ascent v2.6 software (Thermo Fisher Scientific SLU, Barcelona, Spain) according to the concentrations of the standard.

Plasma concentration of the Ig (IgG1, IgG2a, IgG2b, IgG2c, IgM, and IgA) and the cytokines (interleukin (IL) IL-1α, IL-1β IL-2, IL-4, IL-5, IL-6, IL-10, IL-12 P70, IL-13, IL-17α, interferon (IFN)-γ, granulocyte-macrophage colony-stimulating factor (GM-CSF), granulocyte colony stimulating factor 3 (G-CSF3) and tumor necrosis factor (TNF)-α) were quantified using ProcartaPlex™ Multiplex immunoassays (Thermo Fisher Scientific, Barcelona, Spain) as described in previous studies (26), in which specific color-coded capture beads were bound to the Ig/cytokine of interest. Then, different detection antibodies conjugated to phycoerythrin were added. The specific concentration of each analyte was obtained by MAGPIX^®^ analyzer (Luminex Corporation, Austin, TX, United States) at the Scientific and Technological Centers of the University of Barcelona (CCiT-UB). The lower limits of detection of the assay were as follows: 0.15 ng/mL for IgM; 1.15 ng/mL for IgG1; 2.08 ng/mL for IgG2a; 2.67 ng/mL for IgG2b; 4.21 pg/mL for IgG2c; 0.46 pg/mL for IgA; 10.49 pg/mL for IL-1α; 12.96 pg/mL for IL-1β; 1.81 pg/mL for IL-2, 0.62 pg/mL for IL-4; 1.41 pg/mL for IL-5; 2.18 pg/mL for IL-6; 6.00 pg/mL for IL-10; 4.22 pg/mL for IL-12p70; 3.00 pg/mL for IL-13; 3.34 pg/mL for IFN-γ; 4.81 pg/mL for GM-CSF; 4.90 pg/mL for G-CSF3, and 2.88 pg/mL for TNF- α.

### Immunofluorescence Staining and Flow Cytometry Analysis

The spleen cells were isolated as previously described (27). Then, cell number and *via*bility were determined using an automated cell counter (Countess™, Invitrogen, Madrid, Spain) by staining dead cells with trypan blue.

Spleen cells (5 × 10^5^ cells) were stained using immunofluorescence techniques. The mouse anti-rat monoclonal antibody conjugated to fluorescein isothiocyanate, phycoerythrin, peridinin chlorophyll protein, allophycocyanin, or brilliant violet 421 used here were anti-TCRαβ (R73), anti-TCRγδ (V65), anti-CD4 (OX-35), anti-CD8α (OX-8), and anti-NKR-P1A (10/78), all from BD Biosciences (San Diego, CA, United States); anti-CD45RA (OX-33) from Caltag (Burlingame, CA, United States); anti-CD8β (3⋅41) from Serotec (Kidlington, Oxford, United Kingdom); anti-CD25 (OX-39), anti-αE integrin (OX-62) and anti-CD62L (OX-85) from BioLegend (San Diego, CA, United States).

For cell subset differentiation four different antibody panels were used. Panel 1: CD25/CD45RA/CD8α/CD4; panel 2: αE integrin/CD62L/CD8α/CD4/CD45RA; panel 3: TCRαβ/NK/CD8α/CD4 and panel 4: CD8α/CD8β/TCRγδ. The first panel allowed to identify the activated B and T cells. B cells (CR45RA + cells, which is a B cell marker in rats) were identified in the second panel. In the panel 3 NK cells (TCRαβ-NK +) could be differentiated from NKT cells (TCRαβ + NK +) and T cells (TCRαβ + NK- cells from panel 3 in combination with the TCRγδ + cells from panel 4). From the panel 3, the proportion of Th cells (CD4 + CD8-TCRαβ + NK- cells) was obtained. Moreover, the CD8 + TCRαβ + NK- cells (panel 3) plus the TCRγδ + cells (panel 4) could be considered as Tc cells. The αE integrin/CD62L pattern in B, Th and Tc cells were studied in the panel 2. Results are expressed as the proportion of positive cells for a certain marker in each particular subset determined by the combination of other markers with respect to the total lymphocytes gated. Staining was developed following procedures described in previous studies (28), and samples were analyzed using a Gallios™ flow cytometer (Beckman Coulter Inc., Madrid, Spain) at the cytometry service of the Scientific and Technological Centres of the University of Barcelona (CCiT-UB). The obtained data were analyzed with FlowJo software (Tree Star Inc., Ashland, OR, United States).

### Gene Expression Analysis

On days 21 and 42, a 1 cm of a central portion of the small intestine was homogenized for 30 s in lysing matrix tubes (MP Biomedicals, Illkirch, France) using a FastPrep-24 instrument (MP Biomedicals), as previously described (29). After RNA isolation with the RNeasy^®^ Mini Kit (Qiagen, Madrid, Spain) its purity and concentration were determined with a NanoPhotometer (BioNova Scientific S.L., Fremont, CA, United States). Later, the corresponding cDNA was obtained using the thermal cycler PTC-100 Programmable Thermal Controller and TaqMan^®^ Reverse Transcription Reagents (Applied Biosystems, AB, Weiterstadt, Germany).

The specific PCR TaqMan^®^ primers (AB) used to assess gene expression with real-time PCR (ABI Prism 7900 HT, AB) were directed to the detection of free fatty acid receptor 2 (Ffar2) [Rn02345824_s1, inventoried (I)], encoding for G-protein-coupled receptor 43 (Gpr43), barrier function molecules such as mucin (Muc)2 (Rn01498206_m1, I), Muc3 (Rn01481134_m1, I), ocludin (Ocln, Rn00580064_m1, I), claudin (Cldn)2 (Rn02063575_s1, I) and Cldn4 (Rn01196224_s1, I) as well as to Toll-like Receptors (TLR), such as TLR2 (Rn02133647_s1, I), TLR4 (Rn00569848_m1, I), TLR5 (Rn04219239_s1, I), and TLR9 (Rn01640054_m1, I), and maturation markers such as Fc fragment of IgG receptor and transporter (Fcgrt) [Rn00583712_m1, I, encoding for neonatal Fc receptor (FcRn)]. The relative gene expression was normalized to the housekeeping gene glucuronidase beta (Gusb) (Rn00566655_m1, I) using the 2^–ΔΔCt^ method (30). Results are expressed as the percentage of expression in each experimental group normalized to the mean value obtained for the REF group, which was set at 100%, as in previous studies (18).

### Statistical Analysis

Results are expressed as mean ± SEM. The Statistical Package for Social Sciences (SPSS v22.0) (IBM, Chicago, IL, United States) was used for statistical analysis. Data was tested for homogeneity of variance and normality distribution by Levene’s and Shapiro-Wilk tests, respectively. When data was homogeneous and had a normal behavior, conventional one-way ANOVA test was carried out followed by the *post hoc* Bonferroni. Otherwise, the non-parametric Kruskal-Wallis test followed by the *post hoc* Mann-Whitney U test was performed. To study the effect of the overload of *S. epidermidis* in time-dependent variables, two-way ANOVA followed by the *post hoc* Bonferroni was assessed. Significant differences were established when *p* < 0.05.

## Results

### Growth and Morphometry

**FIGURE 1:**
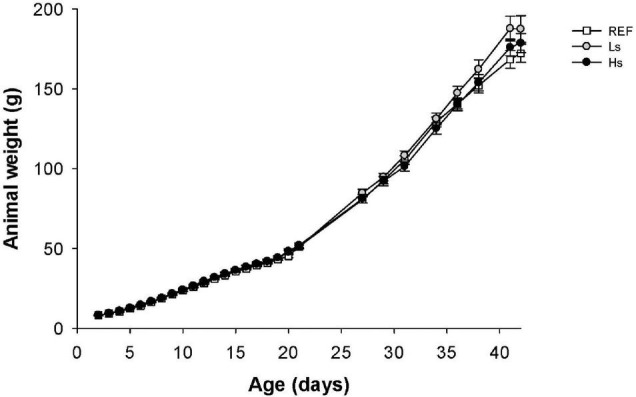
Body weight (g) of suckling rats during the study (from days 2 to 42 of life). Results are expressed as mean ± SEM (*n* = 24 animals/group). REF, reference group; Ls, group supplemented with low dose of *S. epidermidis* and Hs, group supplemented with high dose of *S. epidermidis*.

The body weight of pups was measured daily from days 2 to 21 and weekly from days 22 to 42 of life (Figure 1). The supplementation with the low and high doses of *S. epidermidis* did not have an influence on the normal growth observed in the REF group.

Moreover, data from male and female were also studied separately to analyze if the intervention influenced differently in both sexes (Supplementary Figure 1). No differences between sexes were observed in the first study period (d2–d21) in the same group or between males and females from different groups. Only, in the Ls group, the body weight of males was higher than that of females in the last week of the study (*p* < 0.05).

With regard to morphometry (Supplementary Table 1), the bacterial overload during suckling did not affect any of the variables analyzed at day 21, however, the Ls and Hs groups showed higher naso-anal length than REF animals at day 42. Moreover, no differences were observed in the organs’ weights between groups either at day 21 or at day 42. Morphometry and organs’ weight variables were influenced by the age (*p* < 0.05), regardless the type of intervention, with the exception of the small intestine body weight ratio, which was not affected.

Moreover, as it can be observed in the Table 1, some alterations in the hematological variables due to the bacterial overload were induced. On day 21, Hs group showed higher counts of white blood cells (WBC) and lymphocytes compared to REF and Ls animals. Moreover, the counts of monocytes (MID) in Hs group were higher than those in Ls. On day 42, hemoglobin (HBG) concentration and mean corpuscular hemoglobin (MCH) in Ls supplemented animals were lower than in the REF group. Surprisingly these changes were not observed in Hs animals, which showed higher levels of WBC compared to REF animals due to an increase in lymphocytes, MID and granulocytes (GRAN). In addition, the mean platelet volume (MPV) in Hs group was lower than in REF group.

**TABLE 1:** Hematological variables at the end of the supplementation (day 21 of life) and 3 weeks after (day 42 of life; the end of the study).

	REF	Ls	Hs
**Day 21**			
WBC (10^9^ cells/L)	5.3 ± 0.7	4.8 ± 0.5	**7.5 ± 1.0** *^#^
LYM (%)	69.1 ± 6.7	68.9 ± 2.6	69.8 ± 1.1
MID (%)	8.0 ± 0.8	13.5 ± 5.3	7.9 ± 0.4
GRAN (%)	22.9 ± 2.4	23.0 ± 2.2	22.3 ± 1.0
LYM (10^9^ cells/L)	3.6 ± 0.5	3.3 ± 0.4	**5.2 ± 0.7** *^#^
MID (10^9^ cells/L)	0.4 ± 0.1	0.3 ± 0.0	0.5 ± 0.1^#^
GRAN (10^9^ cells/L)	1.5 ± 0.2	1.2 ± 0.2	1.8 ± 0.3
HGB (g/L)	85.1 ± 8.2	84.9 ± 1.2	83.4 ± 2.2
HCT (%)	23.9 ± 2.3	24.1 ± 0.6	24.8 ± 1.0
MCV (fL)	61.6 ± 5.9	61.1 ± 0.5	62.0 ± 2.0
MCH (pg)	21.9 ± 2.1	21.6 ± 0.6	20.8 ± 0.3
PLT (10^9^ cells/L)	486.4 ± 52.1	521.6 ± 34.5	522.4 ± 51.4
MPV (fL)	8.5 ± 0.8	8.5 ± 0.2	8.9 ± 0.2
**Day 42**			
WBC (10^9^ cells/L)	6.6 ± 0.8	6.6 ± 0.6 ^δ^	**8.0 ± 0.5** *
LYM (%)	76.2 ± 1.4 ^δ^	74.8 ± 2.3	75.5 ± 1.2 ^δ^
MID (%)	7.2 ± 0.4	7.6 ± 0.4	7.1 ± 0.3
GRAN (%)	16.6 ± 1.0 ^δ^	17.6 ± 2.0	17.4 ± 1.1 ^δ^
LYM (10^9^ cells/L)	4.9 ± 0.4 ^δ^	4.9 ± 0.5 ^δ^	**6.0 ± 0.4** *
MID (10^9^ cells/L)	0.4 ± 0.1	0.5 ± 0.1	**0.5 ± 0.0** *
GRAN (10^9^ cells/L)	1.2 ± 0.2	1.2 ± 0.2	**1.5 ± 0.1** *
HGB (g/L)	125.2 ± 1.4 ^δ^	**118.0 ± 1.9** * ^δ^	121.5 ± 1.4 ^δ^
HCT (%)	34.8 ± 0.7 ^δ^	34.6 ± 0.7 ^δ^	34.1 ± 0.6 ^δ^
MCV (fL)	60.0 ± 0.5	60.4 ± 0.8	60.5 ± 0.5
MCH (pg)	21.6 ± 0.4	**20.6 ± 0.2** *	21.5 ± 0.4 ^#^
PLT (10^9^ cells/L)	470.6 ± 40.1	469.9 ± 37.6	526.0 ± 44.5
MPV (fL)	9.5 ± 0.7	9.4 ± 0.7	**8.3 ± 0.2** *

White Blood Cells (WBC), lymphocyte (LYM), monocytes, eosinophils, basophils, blasts, and other precursor white cells (MID), neutrophils, monocytes, eosinophils, and basophils (GRAN), hemoglobin (HGB), hematocrit (HCT), Mean Cell Volume (MCV), Mean Cell Hemoglobin (MCH), platelets (PLT) and Mean Platelet Volume (MPV). Results are expressed ad mean ± SEM (n = 10). Statistical significance: *p < 0.05 vs. REF and ^#^p < 0.05 Hs vs. Ls and ^δ^day 42 vs. day 21. REF, reference group; Ls, group supplemented with low dose of S. epidermidis and Hs, group supplemented with high dose of S. epidermidis. Bold values mean statistical difference with respect to REF group.

Comparing 21- and 42-day-old animals it can be observed that the number of LYM, HGB concentration, and HCT increase with age. These age-associated changes were also present in Ls and Hs animals with the exception of LYM on day 42 which were similar to those observed on day 21.

### Fecal and Cecal Content Variables

**FIGURE 2:**
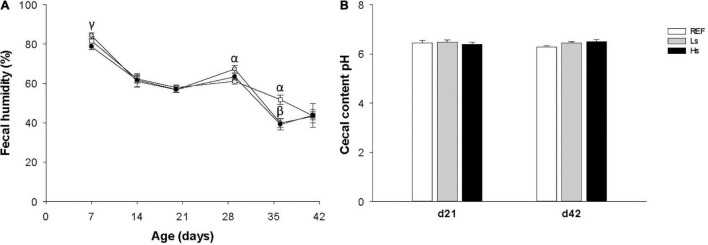
Fecal humidity during the study (from days 7 to 42 of life) **(A)** and cecal content pH on day 21 (the end of the supplementation) and day 42 (3 weeks after the end of the supplementation) **(B)**. Results are expressed as mean ± SEM (*n* = 12–24 animals/group). Statistical differences: ^α^*p* < 0.05 Ls vs. REF, ^β^*p* < 0.05 Hs vs. REF and ^γ^*p* < 0.05 Hs vs. Ls. REF, reference group; Ls, group supplemented with low dose of *S. epidermidis* and Hs, group supplemented with high dose of *S. epidermidis*.

Fecal humidity was analyzed weekly and cecal pH was determined on days 21 and 42 in order to evaluate whether the continuous administration of *S. epidermidis* during suckling influenced the intestinal environment (Figure 2). During the administration period a small increase in fecal humidity in the Ls group was observed on day 7 with respect to Hs group, afterward no changes were observed on days 14 and 21 (Figure 2A). One week after weaning (d28) and without the bacterial overload supplementation, the humidity of Ls group’s feces was higher than that of the REF group. Then, on day 35 both Ls and Hs groups had lower fecal humidity than REF one. Finally, on day 42 no differences between groups were observed. With regard to cecal content pH, similar values were observed between groups on days 21 and 42 of life suggesting no effect of the *S. epidermidis* overload on this variable (Figure 2B).

### Immunoglobulin and Cytokine Plasma Levels

**FIGURE 3:**
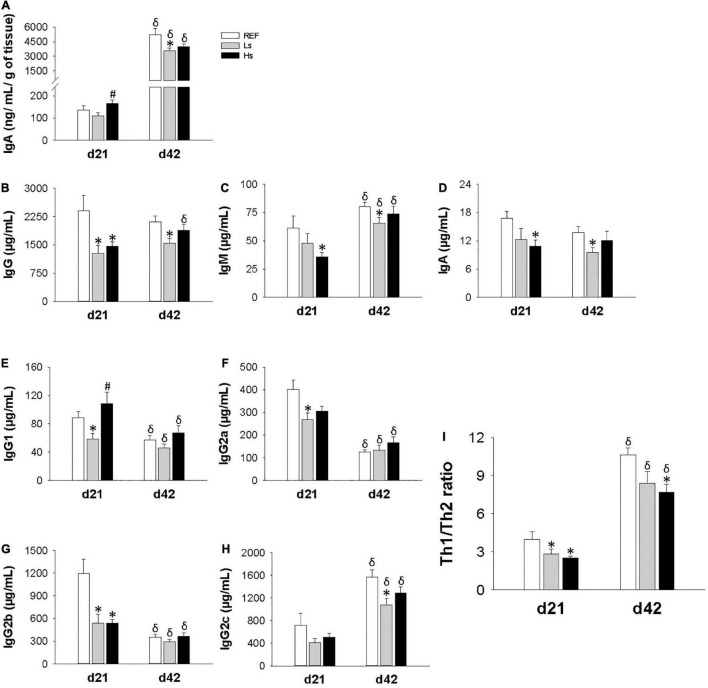
Immunoglobulin concentration in different compartments at the end of the supplementation (day 21 of life) and 3 weeks after (day 42 of life; the end of the study). Intestinal IgA **(A)**, and plasma levels of Ig isotypes **(B–H)**, Th1/Th2 ratio **(I)** refers to the relationship between Th1 associated Ig (IgG2b + IgG2c) and Th2 associated Ig (IgG1 + IgG2a). Results are expressed as mean ± SEM (*n* = 12 animals/group). Statistical differences: *p < 0.05 vs. REF, ^#^*p* < 0.05 Hs vs. Ls and ^δ^ day 42 vs. day 21. REF, reference group; Ls, group supplemented with low dose of *S. epidermidis* and Hs, group supplemented with high dose of *S. epidermidis*.

Intestinal IgA and plasma concentrations of IgG, IgM, and IgA isotypes, IgG subclasses, as well as the Th1/Th2 ratio were quantified at the end of the supplementation (day 21 of life) and 3 weeks after (day 42 of life; the end of the study) (Figure 3). On day 21, the intestinal levels of IgA in both supplemented groups were similar to the control one, however, the highest dose induced higher increase in IgA with respect to the low dose (*p* < 0.05) (Figure 3A). On the contrary, on day 42, a lower intestinal IgA concentration was found in Ls group compared to REF one (*p* < 0.05). Intestinal IgA levels were higher at day 42 than at day 21 for all the groups of study (*p* < 0.05) independently of the intervention.

Important changes were observed in plasma Ig concentrations (Figure 3 and Supplementary Table 2). On day 21, IgG concentration in Ls animals was lower than in REF ones whereas in Hs animals all three isotypes levels (IgG, IgM, and IgA) were lower in Hs group than in REF group (Figures 3B–D, respectively). It can also be noted that age associated changes were only found for IgM, but not for IgA or IgG.

With regard to IgG subclasses (Figures 3E–H), on day 21 IgG1, IgG2a, and IgG2b levels were lower in Ls group than in REF animals, whereas only a significant reduction in IgG2b was observed in Hs group. On day 42, the previous changes were not maintained and only a reduction in IgG2c was observed in Ls group with respect to REF group on day 42. Overall, the changes observed in the IgG subclasses led to a decreased Th1/Th2 ratio in both Ls and Hs groups on day 21 and in Hs on day 42.

In addition, although IgG levels are constant through age a reduction in IgG1, IgG2a, and IgG2b and an increase in IgG2c can be observed with age. These changes lead to an increase in Th1/Th2 ratio associated to age.

Furthermore, plasma cytokine levels were also measured at the end of the supplementation (day 21 of life) and 3 weeks after (day 42 of life; the end of the study). Neither the age of the animals nor the bacterial overloads were conditions able to induce changes in the plasmatic cytokine pattern (Table 2).

**TABLE 2:** Plasma cytokines concentration at the end of the supplementation (day 21 of life) and 3 weeks after (day 42 of life).

Cytokine (pg/mL)	REF	Ls	Hs
**Day 21**			
G-CSF3	4.4 ± 2.2 (100%)	6.6 ± 3.1 (80%)	5.1 ± 1.7 (80%)
IL-1-α	62.1 ± 62.1 (10%)	120.9 ± 113.7 (40%)	21.7 ± 14.6 (20%)
IL-10	101.3 ± 93.8 (30%)	189.0 ± 131.5 (60%)	125.0 ± 53.0 (60%)
IL-6	0.0 ± 0.0 (0%)	1.2 ± 1.2 (10%)	0.0 ± 0.0 (0%)
IL-1-β	162.8 ± 54.5 (70%)	48.2 ± 20.5 (80%)	47.2 ± 21.5 (80%)
IL-2	30.0 ± 11.5 (70%)	7.4 ± 3.7 (70%)	6.0 ± 2.6 (70%)
IL-4	0.80 ± 0.4 (100%)	1.0 ± 0.6 (60%)	1.0 ± 0.4 (60%)
IFN- γ	2.7 ± 1.5 (70%)	3.7 ± 2.4 (70%)	6.5 ± 2.9 (60%)
IL-5	2.3 ± 2.0 (50%)	5.3 ± 3.6 (50%)	2.3 ± 1.0 (50%)
IL-13	34.1 ± 17.1 (60%)	7.6 ± 4.2 (80%)	5.3 ± 3.0 (70%)
IL-12p70	48.4 ± 42.7 (80%)	14.0 ± 7.8 (80%)	20.3 ± 9.2 (90%)
GM-CSF	1.2 ± 1.2 (10%)	2.8 ± 2.8 (10%)	0.0 ± 0.0 (0%)
TNF-α	12.2 ± 4.2 (70%)	3.1 ± 1.5 (60%)	3.3 ± 1.4 (70%)
IL-17-α	2.0 ± 1.6 (30%)	4.8 ± 2.4 (60%)	2.3 ± 0.8 (60%)
**Day 42**			
G-CSF3	7.4 ± 5.1 (80%)	7.4 ± 4.6 (100%)	3.6 ± 0.9 (100%)
IL-1-α	177.7 ± 164.5 (20%)	177.7 ± 164.5 (20%)	13.8 ± 12.2 (30%)
IL-10	237.6 ± 206.0 (50%)	265.2 ± 199.0 (50%)	79.7 ± 37.3 (70%)
IL-6	1.7 ± 1.7 (10%)	1.6 ± 1.6 (10%)	0.0 ± 0.0 (0%)
IL-1-β	93.06 ± 25.5 (70%)	202.0 ± 105.7 (70%)	50.6 ± 18.3 (70%)
IL-2	11.9 ± 4.1 (80%)	29.7 ± 13.8 (70%)	6.5 ± 1.9 (90%)
IL-4	1.5 ± 1.1 (80%)	1.5 ± 0.9 (100%)	0.7 ± 0.2 (100%)
IFN- γ	4.4 ± 3.5 (50%)	4.5 ± 3.4 (70%)	1.5 ± 0.6 (80%)
IL-5	5.5 ± 4.2 (50%)	5.8 ± 4.2 (80%)	2.6 ± 0.7 (100%)
IL-13	10.3 ± 4.5 (80%)	23.4 ± 8.9 (70%)	7.1 ± 1.7 (90%)
IL-12p70	11.7 ± 8.4 (70%)	11.6 ± 7.0 (100%)	4.0 ± 1.5 (100%)
GM-CSF	4.4 ± 4.4 (10%)	4.1 ± 4.1 (10%)	0.0 ± 0.0 (0%)
TNF-α	6.5 ± 1.8 (70%)	11.8 ± 5.5 (70%)	2.5 ± 1.0 (60%)
IL-17-α	4.7 ± 3.4 (50%)	5.1 ± 3.0 (70%)	8.6 ± 6.6 (70%)

*Interleukin (IL), interferon (IFN)-γ, granulocyte-macrophage colony-stimulating factor (GM-CSF), granulocyte colony stimulating factor 3 (G-CSF3) and tumor necrosis factor (TNF)-α. Percentage of detection is represented in parenthesis. Results are expressed as mean ± SEM (n = 10).*

### Phenotype of Spleen Cells

At days 21 and 42 of life, spleen cells were isolated to evaluate the influence of the overload of *S. epidermidis* on the phenotype of lymphocytes in terms of main subsets proportion, homing receptors and activation status. The percentages of the main cell types are summarized in Table 3. B, T, Th, Tc, and NKT cell proportions were similar between groups at days 21 and 42.

**TABLE 3:** Main spleen lymphocyte populations at the end of the supplementation (day 21 of life) and 3 weeks later (day 42 of life).

	REF	Ls	Hs
**Day 21**			
B cells (CD45RA^+^)	60.88 ± 2.29	60.52 ± 2.37	65.01 ± 1.87
T cells (TCRαβ^+^ NK^–^ y TCRγδ^+^)	18.63 ± 1.79	20.93 ± 2.35	15.62 ± 1.28
Th cells (CD4^+^ CD8^–^TCRαβ^+^)	12.19 ± 1.18	13.77 ± 1.68	10.00 ± 0.83
Tc cells (CD8^+^ TCRαβ^+^ NK^–^ y TCRγδ^+^)	5.21 ± 0.57	5.86 ± 0.61	4.57 ± 0.49
T cells TCRαβ^+^ (CD8^+^ TCRαβ^+^ NK^–^)	4.27 ± 0.49	4.89 ± 0.58	3.69 ± 0.42
T cells TCRγδ^+^	0.94 ± 0.10	0.97 ± 0.10	0.88 ± 0.08
CD8^–^ (%)	21.75 ± 2.65	25.51 ± 1.57	31.84 ± 2.83 *
CD8^+^ (%)	78.25 ± 2.65	74.49 ± 1.57	68.16 ± 2.83 *
CD8αα^+^ (%)	51.53 ± 2.50	47.36 ± 2.07	52.06 ± 2.39
CD8αβ^+^ (%)	48.47 ± 2.50	52.64 ± 2.07	47.94 ± 2.39
NKT cells (TCRαβ^+^NK^+^)	3.50 ± 0.39	3.59 ± 0.37	3.01 ± 0.26
NK cells (TCRαβ- NK^+^)	8.00 ± 0.86	7.32 ± 0.68	6.94 ± 0.62
CD8^–^ (%)	27.06 ± 2.03	24.21 ± 1.45	29.39 ± 2.17
CD8^+^ (%)	72.94 ± 2.03	75.79 ± 1.45	70.61 ± 2.17
CD8αα^+^ cells	4.84 ± 0.50	5.23 ± 0.70	4.49 ± 0.36
CD8αβ^+^ cells	6.13 ± 0.86	6.44 ± 0.57	4.83 ± 0.42
Ratio CD8αα/CD8αβ	0.84 ± 0.06	0.81 ± 0.09	1.05 ± 0.15
**Day 42**			
B cells (CD45RA^+^)	48.96 ± 1.13 ^δ^	50.14 ± 1.67 ^δ^	49.58 ± 1.73 ^δ^
T cells (TCRαβ^+^ NK^–^ y TCRγδ^+^)	39.48 ± 1.27 ^δ^	37.27 ± 1.69 ^δ^	35.31 ± 1.76 ^δ^
Th cells (CD4^+^CD8^–^ TCRαβ^+^)	24.61 ± 1.00 ^δ^	22.66 ± 0.98 ^δ^	21.26 ± 1.11 ^δ^
Tc cells (CD8^+^ TCRαβ^+^ NK^–^ y TCRγδ^+^)	13.29 ± 1.43 ^δ^	13.00 ± 0.98 ^δ^	12.24 ± 0.92 ^δ^
T cells TCRαβ^+^ (CD8^+^ TCRαβ^+^ NK^–^)	12.23 ± 0.68 ^δ^	11.83 ± 0.92 ^δ^	11.08 ± 0.88 ^δ^
T cells TCRγδ^+^	1.07 ± 0.07 ^δ^	1.18 ± 0.12 ^δ^	1.16 ± 0.05 ^δ^
CD8^–^ (%)	21.60 ± 1.02	19.00 ± 1.20 ^δ^	19.63 ± 1.55 ^δ^
CD8^+^ (%)	78.40 ± 1.02	81.00 ± 1.20 ^δ^	80.37 ± 1.55 ^δ^
CD8αα^+^ (%)	39.02 ± 1.33 ^δ^	41.44 ± 1.80	46.93 ± 2.75 *
CD8αβ^+^ (%)	60.98 ± 1.33 ^δ^	58.56 ± 1.80	53.07 ± 2.75 *
NKT cells (TCRαβ^+^NK^+^)	3.86 ± 0.20 ^δ^	4.07 ± 0.22 ^δ^	4.00 ± 0.27 ^δ^
NK cells (TCRαβ- NK^+^)	6.97 ± 0.42	7.42 ± 0.43	8.71 ± 0.23 *^#^
CD8^–^ (%)	20.52 ± 0.97 ^δ^	18.35 ± 1.29 ^δ^	17.91 ± 0.97 ^δ^
CD8^+^ (%)	79.48 ± 0.97 ^δ^	81.65 ± 1.29 ^δ^	82.09 ± 0.97 ^δ^
CD8αα^+^ cells	6.77 ± 0.41 ^δ^	6.96 ± 0.64 ^δ^	7.26 ± 0.30 * ^δ^
CD8αβ^+^ cells	12.68 ± 0.80 ^δ^	12.23 ± 1.39 ^δ^	9.87 ± 0.96 *^δ^
Ratio CD8αα/CD8αβ	0.55 ± 0.05 ^δ^	0.60 ± 0.06 ^δ^	0.81 ± 0.08 * ^δ^

Results are expressed as the percentage of total lymphocytes (mean ± SEM) (n = 12 animals/group). Statistical significance: *p < 0.05 vs. REF, ^#^Hs vs. Ls and ^δ^day 42 vs. day 21. REF, reference group; Ls, group supplemented with low dose of S. epidermidis and Hs, group supplemented with high dose of S. epidermidis.

However, T TCRγδ^+^ cells displayed some differences. Although no changes were observed in total T TCRγδ^+^ cells, at day 21, a higher proportion of T TCRγδ^+^ CD8^–^ cells and a lower proportion of T TCRγδ^+^ CD8^+^ cells were found in Hs group compared to REF group (*p* < 0.05). Furthermore, at day 42, a higher percentage of T TCRγδ^+^ CD8αα^+^ cells and a lower percentage of T TCRγδ^+^ CD8αβ^+^ cells were observed in Hs animals compared to REF group (*p* < 0.05). Moreover, even though no changes were observed in the proportion of NK cells at day 21, at day 42 it was higher in Hs group compared to REF and Ls animals (*p* < 0.05). In addition to this, only at 42 days, the proportion of CD8αβ^+^ cells was lower, thus resulting in an increase of CD8αα^+^ cells percentage and CD8αα/CD8αβ ratio in Hs group compared to REF animals (*p* < 0.05).

The main subsets (B, T, Th, Tc, and NKT) were influenced by age (by comparing day 42 with day 21) in all groups with the exception of NK cells, which did not change. In addition, some differences associated to age in particular groups also appeared. In Ls and Hs T TCRγδ^+^ CD8^–^ and T TCRγδ^+^ CD8^+^ cell proportions were reduced and increased, respectively, at day 42 compared to day 21. The proportion of T TCRγδ^+^ CD8αα^+^ cells and T TCRγδ^+^ CD8αβ^+^ were also reduced and increased, respectively, only in REF animals at day 42 compared to day 21 (*p* < 0.05).

**FIGURE 4:**
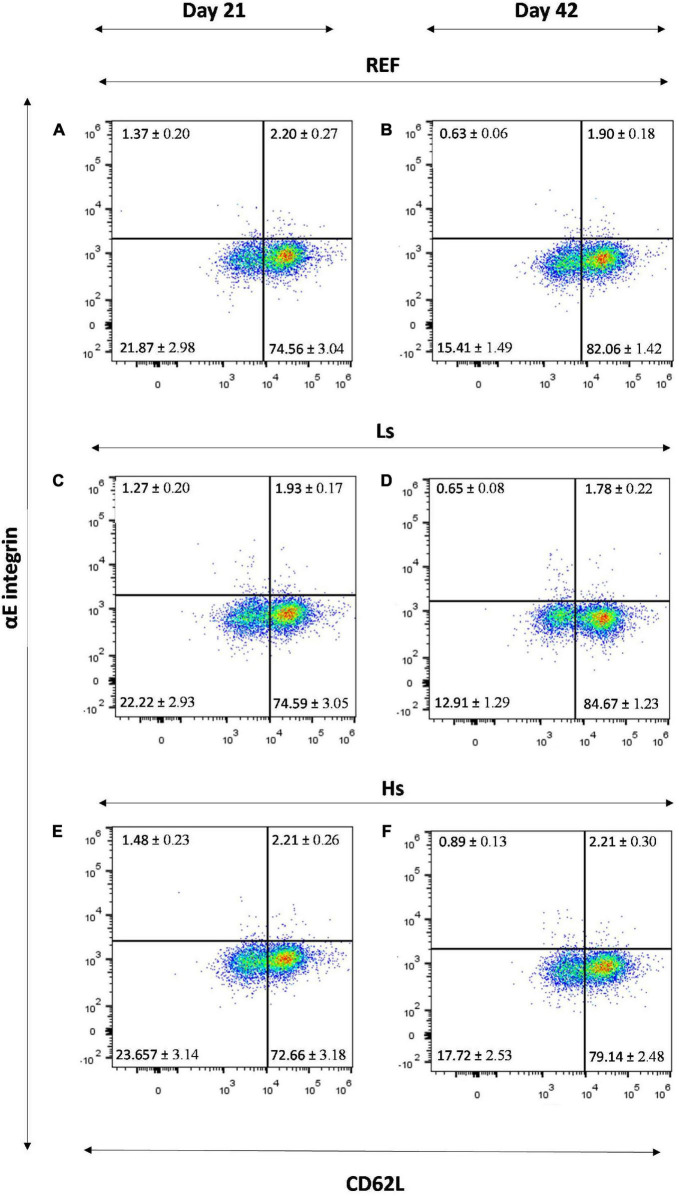
αE integrin/CD62L molecular pattern in the total lymphocytes of the spleen of REF **(A)**, Ls **(C)**, Hs **(E)** animals at day 21 and REF **(B)**, Ls **(D)**, Hs **(F)** animals at day 42. Representative histograms for each group. Results are expressed as mean ± S.E.M (*n* = 12 animals/group). REF, reference group; Ls, group supplemented with low dose of *S. epidermidis* and Hs, group supplemented with high dose of *S. epidermidis*.

The αE integrin/CD62L molecular pattern was measured in the total lymphocytes (Figure 4) and in the B, Th and Tc subsets (Table 4). In the total lymphocytes, the αE integrin/CD62L pattern was similar between groups at days 21 and 42, being most of the cells αE^–^CD62L^+^ (Figure 4).

**TABLE 4:** Expression of the integrin αE and the selectin CD62L in Th, Tc, and B lymphocytes in the spleen at the end of the supplementation (day 21 of life) and 3 weeks later (day 42 of life).

	REF	Ls	Hs
**Day 21**			
**α E^+^CD62L^–^**			
B cells (CD45RA^+^)	0.05 ± 0.01	0.05 ± 0.01	0.04 ± 0.01
Th cells CD4^+^CD8^–^	1.88 ± 0.31	1.90 ± 0.32	2.41 ± 0.38
Tc cells CD4^–^CD8^+^	2.17 ± 0.23	1.85 ± 0.30	1.99 ± 0.26
**α E^+^CD62L^+^**			
B cells (CD45RA^+^)	2.30 ± 0.32	2.28 ± 0.34	2.17 ± 0.36
Th cells CD4^+^CD8^–^	1.12 ± 0.12	1.50 ± 0.26	1.54 ± 0.33
Tc cells CD4^–^CD8^+^	3.19 ± 0.62	2.01 ± 0.24	2.93 ± 0.57
**α E** ^–^ **CD62L^+^**			
B cells (CD45RA^+^)	95.66 ± 0.41	95.84 ± 0.32	95.74 ± 0.28
Th cells CD4^+^CD8^–^	60.44 ± 4.02	61.35 ± 3.12	55.69 ± 3.40
Tc cells CD4^–^CD8^+^	67.58 ± 2.99	66.57 ± 4.04	66.55 ± 2.60
**α E–CD62L–**			
B cells (CD45RA^+^)	1.99 ± 0.33	1.82 ± 0.35	2.04 ± 0.27
Th cells CD4^+^CD8^–^	36.57 ± 3.81	35.26 ± 2.94	40.38 ± 3.67
Tc cells CD4^–^CD8^+^	27.05 ± 3.51	29.57 ± 3.89	28.53 ± 3.25
**Day 42**			
**α E^+^CD62L^–^**			
B cells (CD45RA^+^)	0.24 ± 0.06 ^δ^	0.19 ± 0.03 ^δ^	0.32 ± 0.10 ^δ^
Th cells CD4^+^CD8^–^	0.89 ± 0.08 ^δ^	1.07 ± 0.12 ^δ^	1.27 ± 0.22 ^δ^
Tc cells CD4^–^CD8^+^	0.76 ± 0.09 ^δ^	0.68 ± 0.10 ^δ^	1.10 ± 0.23 ^δ^
**α E^+^CD62L^+^**			
B cells (CD45RA^+^)	3.00 ± 0.31 ^δ^	2.92 ± 0.47 ^δ^	4.03 ± 0.58 ^δ^
Th cells CD4^+^CD8^–^	1.15 ± 0.10	1.26 ± 0.20	0.98 ± 0.18
Tc cells CD4^–^CD8^+^	1.54 ± 0.17 ^δ^	1.17 ± 0.17 ^δ^	1.62 ± 0.18 ^δ^
**α E** ^–^ **CD62L^+^**			
B cells (CD45RA^+^)	92.83 ± 0.57 ^δ^	92.48 ± 0.58 ^δ^	91.67 ± 0.97 ^δ^
Th cells CD4^+^CD8^–^	75.64 ± 1.22 ^δ^	77.54 ± 0.94 ^δ^	68.45 ± 4.17 ^δ^
Tc cells CD4^–^CD8^+^	77.72 ± 1.23 ^δ^	83.98 ± 1.47 * ^δ^	77.31 ± 3.90 ^δ^
**α E** ^–^ **CD62L^–^**			
B cells (CD45RA^+^)	3.93 ± 0.36 ^δ^	4.42 ± 0.42 ^δ^	3.92 ± 0.44 ^δ^
Th cells CD4^+^CD8^–^	22.32 ± 1.19 ^δ^	20.15 ± 0.84 ^δ^	29.29 ± 4.07 ^#^ ^δ^
Tc cells CD4^–^CD8^+^	19.99 ± 1.25	14.18 ± 1.61 * ^δ^	19.96 ± 3.79 ^δ^

Results are expressed as the percentage of total lymphocytes (mean ± SEM) (n = 12 animals/group). Statistical significance: *p< 0.05 vs. REF, ^#^Hs vs. Ls and ^δ^day 42 vs. day 21. REF, reference group; Ls, group supplemented with low dose of S. epidermidis and Hs: group supplemented with high dose of S. epidermidis.

When these adhesion markers were studied in more specific subsets, as it can be observed in Table 4, some changes were found due to the *S. epidermidis* administration only at day 42. Cells from Ls group showed a higher percentage of Tc cells with αE^–^CD62L^+^ phenotype and a lower proportion of Tc cells with the αE^–^CD62L^–^ pattern compared to those found in the REF group (p < 0.05). A higher percentage of Th cells with αE^–^CD62L^–^ characteristics were also found in Hs animals compared to Ls group (*p* < 0.05).

Age-associated changes were also found in the αE integrin and the CD62L selectin pattern of B, Th, and Tc cells in all groups except for the Tc αE^–^CD62L^–^ cell proportion, in which no age-associated modification was found in REF animals and Th αE^+^CD62L^+^ which did not change in any group either.

Finally, the expression of the CD25 in Th, Tc and B lymphocytes was measured in the spleen cells on days 21 and 42 (Table 5). No influence of the bacterial overload was found either at day 21 or at day 42. Interestingly, the age associated increase of CD25^+^ Tc cell proportion, and its consequent reduction in that of the CD25^–^ Tc subset, was found in all groups, although an interaction (group × age) was only found in the Hs group (*p* = 0.048).

**TABLE 5:** Expression of the CD25 in Th, Tc and B lymphocytes in the spleen at the end of the supplementation (day 21 of life) and 3 weeks later (day 42 of life).

	REF	Ls	Hs
**Day 21**			
**CD25^+^**			
B cells (CD45RA^+^)	0.27 ± 0.04	0.49 ± 0.17	0.33 ± 0.06
Th cells CD4^+^CD8^–^	7.30 ± 0.50	6.30 ± 0.31	6.85 ± 0.64
Tc cells CD4^–^CD8^+^	0.37 ± 0.02	0.34 ± 0.03	0.32 ± 0.04
**Day 42**			
**CD25^+^**			
B cells (CD45RA^+^)	0.42 ± 0.09	0.26 ± 0.07	0.30 ± 0.06
Th cells CD4^+^CD8^–^	5.73 ± 0.24	5.41 ± 0.33	6.05 ± 0.30
Tc cells CD4^–^CD8^+^	0.35 ± 0.02 ^δ^	0.42 ± 0.03 ^δ^	0.44 ± 0.03 ^δ^

*Results are expressed as the percentage of total lymphocytes (mean ± SEM) (n = 12 animals/group). Statistical significance: ^δ^p < 0.05 day 42 vs. day 21. REF, reference group; Ls, group supplemented with low dose of S. epidermidis and Hs, group supplemented with high dose of S. epidermidis.*

### Intestinal Gene Expression

**FIGURE 5:**
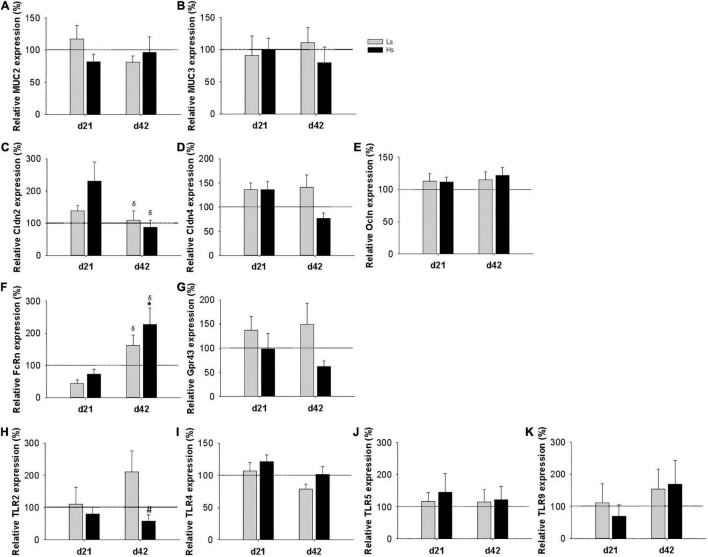
Intestinal gene expression of mucin (MUC) 2 **(A)** and MUC 3 **(B)**, claudin (Cldn) 2, Cldn4 (**C,D**, respectively), occludin (Ocldn) **(E)**, FcRn **(F)**, Gpr43 **(G)**, and Toll like receptors (TLR) 2 **(H)**, TLR4 **(I)**, TLR5 **(J)**, TLR9 **(K)** at days 21 and 42. Relative gene expression was calculated with respect to REF animals, which corresponded to 100% of transcription. Results are expressed as mean ± S.E.M (*n* = 9 animals/group). Statistical significance: *p < 0.05 vs. REF, ^#^ vs. Ls. REF and ^δ^day 42 vs. day 21: reference group; Ls, group supplemented with low dose of *S. epidermidis* and Hs, group supplemented with high dose of *S. epidermidis*.

The gene expression of different molecules involved in the intestinal barrier function [mucin (MUC) 2, MUC3, claudin (Cldn) 2, Cldn4 and occludin (Ocldn)], intestinal immunity (FcRn and Gpr43), and microbiota-host signaling receptors (TLR 2, 4, 5, and 9) was measured at the end of the intervention (day 21 of life) and 3 weeks after (day 42 of life; the end of the study) (Figure 5). At day 21, no influence of the bacterial overload was found in any of the genes analyzed. At day 42, in Hs group a marked increase in the expression of FcRn was observed, being more than two times higher than that in the REF group (*p* < 0.05) and also the expression of TLR2 was 1.7 times lower compared to Ls (*p* < 0.05). Moreover, the levels of Cldn2 and FcRn showed an age-associated reduction and increase, respectively, in all groups, whereas the rest of the genes studied did not show an age-dependent pattern. The FcRn gene expression was the only one showing an age-intervention interaction (*p* < 0.05).

## Discussion

*S. epidermidis* is the first causal agent of mastitis, which causes the inflammation of the mammary gland in lactating mothers (8). Many studies in humans and animal models have only focused on the impact of this situation in the maternal health or in the microbiota alteration induced in the breast milk due to the drastically predominance of the pathogen and resulting reduction in the rest of the bacterial groups (7). However, very few studies have focused on the impact of this process in the breastfed baby.

As it has been shown that an overload of *S. epidermidis* can be transferred *via* breast milk to the neonate (7) it is plausible to hypothesize that it can affect baby’s health, and particularly, its immune development. For this reason, this study includes a preclinical approach in which two different doses of *S. epidermidis* have been administered to neonatal rats, one reflecting a subclinical mastitis and another one mimicking a clinical mastitis. The possible impact of this bacteria overload on the offspring has been studied at the end of the lactation and also later in time, specifically 3 weeks after the last bacterial administration.

One of the aims of this study was to investigate if the daily overload of *S. epidermidis* during the whole suckling period had an impact on growth. Overall, the interventions did not influence the body or organs weights either at the end of the suckling period or 3 weeks later, and just a punctual effect by the two doses at day 42 was observed in the body length. It can be then suggested that the *S. epidermidis* overload during suckling may affect the development of rats in the long term. In this regard, a study by Griffiths et al. reported lower growth rates in the offspring of dams with clinical mastitis at weaning (31) however this finding is not in agreement with the increase observed at 42 days.

With regard to the hematological variables, some changes due to the bacterial overload were found. In our study, the animals receiving a high dose of *S. epidermidis* displayed leukocytosis at day 21, which persisted until day 42. Infection, stress and trauma are the most common causes of leukocytosis (32). In fact, leukocytosis is a good indicator of an infectious process (33) and most patients at an emergency room with a suspicion of an infection have leukocytosis (34). Neutrophils are the most abundant leukocyte in this context (35) and most of acute bacterial infections cause neutrophilia, but some of them causes lymphocytosis such as the bacteria *Bordetella pertussis*, *Treponema pallidum*, *Bartonella henselae* or the genus *Brucella* (36). There is non-available data about whether neonates whose mother have infectious mastitis also present changes in WBC counts. However, in other bacterial infections alterations in hematological variables in neonates have also been observed (37, 38). Therefore, the leukocytosis observed in the Hs animals suggests that the intervention with an overload of *S. epidermidis* induces systemic changes in WBC, specially increasing the levels of lymphocytes to fight the bacteria overload and control it. However, the process does not seem to be resolved at day 42 as suggested by the higher levels of lymphocytes, monocytes and granulocytes present in Hs group compared to REF animals.

The gastrointestinal tract of the pups received a huge number of the *S. epidermidis* for 21 days, thus allowing us to think that this may have an impact on the intestinal ecosystem. None of the doses assayed of *S. epidermidis* overload affected the fecal humidity during suckling although they reduced the humidity of the feces at certain point after weaning. These changes could be due to a gut imbalance caused by the bacterial overload during suckling. Furthermore, cecal pH was not affected either by the *S. epidermidis* overload. To our knowledge, no data is available in the literature with regard to changes in the fecal humidity, pH or microbiota as a result of maternal infectious mastitis neither in animals nor in humans. However, previous studies from our group reported that suckling rats under an infective process (gastroenteritis caused by rotavirus) showed higher fecal pH (39). On the other hand, healthy suckling rats supplemented with galactooligosaccharides and fructooligosaccharides had intestinal microbiota alterations and changes in fecal consistency but not in fecal pH (19). Therefore, humidity and pH could be correlated with the composition of the intestinal microbiota, thus suggesting a change in the microbiota composition of weaned rats after receiving *S. epidermidis* overload during suckling.

Focusing on the effect of the intervention on the development of the immune system, several aspects were studied, such as the plasma Ig levels and the Th1 or Th2 type of response based on IgG types found. It is well known that in the developing neonatal immune system a Th2 type response is induced when microorganisms are first encountered, while in a mature immune system a correct balance between Th1 and Th2 responses can be found (40). In the present study, at the end of the intervention, Ls showed lower concentration of IgG subclasses that resulted in a lower Th1/Th2 IgG ratio. Moreover, the high dose of *S. epidermidis* overload also reduced the levels of IgG, IgM and IgA, and IgG2b showing again a lower Th1/Th2 ratio. These results suggest that the two interventions induced changes in the immune system development reducing their maturation by means of a delay in the Th1/Th2 balance. However, it has to be taken into account that in this stage to life, the Ig-enriched maternal milk influences the pups’ plasma Ig levels, as observed in previous studies (22). But, on day 42 of life, the quantification of pups’ plasma Ig levels is not affected by their mother, and we still found a bias toward a Th2 response in Hs animals compared to REF group and, in Ls group, the levels of IgG, IgA, and IgM were lower compared to REF animals but, unexpectedly, not in Hs group. Overall, it has to be taken into account that the Th1/Th2 balance discussed here is based on IgG subclasses rather than on cytokine levels.

It has been described that early development of altered gut microbiota modifies the Th1/Th2 response toward a Th2 response (41, 42). Hence, the overload of *S. epidermidis* may affect the intestinal microbiota and, therefore, the immune system Th1/Th2 balance of suckling rats. In future studies it would be necessary to study the composition of the pups’ intestinal microbiota and its relationship with the immune development. This preclinical evidence could be in line with the outcome of a recent clinical trial in which a higher presence of *Staphylococcus* in breast milk and also in infant feces was associated to an increase in the risk of respiratory infections in the infants (43).

To further study the Th2 response found in the animals, the cytokine levels in pups’ plasma were also studied at the end of the intervention (day 21) and 3 weeks after (day 42). No differences were observed between groups. In line with the previous findings, no data was found in the literature concerning cytokine production in newborns fed with breast milk from a mother with infectious mastitis either in humans or in animals. Our animal model did not imply changes in the lactating mother; however, it can be plausible that in a real scenario, the *S. epidermidis* would also have an impact on the mother at the systemic levels and in the breast milk composition. In line with this, some results in cows presenting mastitis can be also of interest. Lactating cows suffering from subclinical mastitis showed higher levels of IL-6 and lower concentration of IL-4 and IL-10 (44). Akhtar et al. found that cow mothers with mastitis had higher mRNA expression levels of TNF-α, IL-1β, and IL-6 (45). These changes could modify the cytokine composition of milk and be transferred to the offspring through breastfeeding and affect their immune system development. Taking all together, we cannot discard that although no changes in proinflammatory cytokines have been found in our intervention, the breast milk of mothers suffering from mastitis could contribute to an altered cytokine profile in the milk and in the baby.

During suckling, phenotypical changes in the systemic immune system of neonatal rats correlate with their maturation (46). NK/NKT and B cells seem to develop earlier than T cells in neonates; which mature later, during the second half of suckling period, in terms of phenotype and proliferative ability (46). In this regard, the interventions only induced small changes in spleen lymphocytes subpopulation from Hs animals such as changes of CD8 in TCRγδ^+^ T cells at day 21 and in CD8αα^+^pattern of TCRγδ^+^ cells and CD8αα/CD8αβ ratio at day 42. In addition, the expression of CD25 (alpha chain of the trimeric IL-2 receptor), which is considered a hall mark of cellular activation that is expressed constitutively on the surface of several lymphocytes (47), was not influenced by the interventions in B, Th and Tc cells. For the study of the intestinal homing, the expression of the adhesion molecules CD62L selectin and αE integrin were measured. The αE integrin (CD103) is a receptor for E-cadherin, its interaction allows the adhesion between epithelial cells and T lymphocytes, but it is also expressed on the cell surface of tissue-resident memory CD8^+^ T cells (48). The CD62L selectin is a cell adhesion molecule that is expressed on most circulating leukocytes (49). Naïve CD8^+^ T cells express CD62L, however effector CD8^+^ T cells lack its expression (50). The two *S. epidermidis* overloads during suckling did not show any change in the homing pattern at the end of the intervention, but, at day 42, the presence of αE^–^CD62L^+^ Tc cells from Ls group was higher than in REF animals. To our knowledge, no information is available regarding αE integrin and CD62L pattern in infants fed with breast milk after an infectious mastitis, however, in lactating cows with mastitis a decreased expression of CD62L has been reported (51, 52). Therefore, our results suggest that Tc cell development is delayed in Ls group, but unexpectedly not in Hs animals. Future studies would be necessary to go in depth in the study of the differential effect of each dose in the pattern of αE and CD62L in Tc cells.

Finally, the intestinal gene expression was measured at days 21 and 42. Although some changes in the molecules involved in the intestinal barrier (MUC2, MUC3, Cldn2, Cldn4, and Ocln), or in the microbiota-host signaling receptors (TLR 2, 4, 5, and 9) were expected, the *S. epidermidis* overload did not have any significant influence on them. However, TLR2, which recognize several staphylococcal lipoproteins among other molecules, has a trend to reduce its expression at day 42. In this regard, a higher expression on day 21 would have been expected, thus the biological significance of such change remains to be elucidated. On the contrary, the levels of FcRn in Hs group were higher on day 42 compared to REF animals. In the proximal small intestine epithelium of neonatal rats, FcRn mediates the transcytosis of the IgG present in breast milk (53). After weaning, immature epithelium is replaced by an adult-type epithelium, which does not express FcRn (53). Therefore, as the reduction of FcRn is associated with intestinal epithelial barrier maturation, the higher expression of FcRn in Hs animals compared to REF group, suggests that the maturation of the small gut is also delayed.

This particular model has allowed us to define the impact of a *S. epidermidis* overload on pups during suckling. However, it has some limitations. For instance, the supplementation was performed during the whole suckling period whereas sometimes the mastitis is just a period within lactation. In addition, as commented before, breast milk composition may change due to the mastitis, so the variations in anti-infectious components such as cytokines and Ig, can also have an influence on pup’s immune development. Finally, and although the precise composition of *Staphylococcus* types during mastitis is not clearly defined, only one bacterial strain was used here. Thus, future research is guaranteed to better dissect the real scenario during mastitis and its impact on the baby.

## Conclusion

The intervention with *S. epidermidis* overload during suckling, mimicking what happens when the infant is fed with breast milk from mothers during a mastitis process, affects the immune system development in short and long term. The doses assayed induce small changes in lymphocyte subpopulations, reduce the plasma Ig levels and delay the Th1/Th2 balance causing a bias toward a Th2 response. The low dose affects Tc cells intestinal homing pattern while the high dose has an impact on the hematological variables causing leukocytosis and lymphocytosis and disrupting the intestinal barrier maturation.

## Data Availability Statement

The original contributions presented in the study are included in the article/Supplementary Material, further inquiries can be directed to the corresponding author/s.

## Ethics Statement

The animal study was reviewed and approved by the Ethical Committee for Animal Experimentation of the University of Barcelona and the Catalonia Government (CEEA/Ref. 308/19 and PAMN/Ref.10542, respectively).

## Author Contributions

MO, MR-L, and FP-C conceived and designed the research and were primary responsibility for the final content. CM-F, MO, ÀF, MC, MR-L, and FP-C carried out the experiments and the data analysis and were involved in the interpretation of the data. CM-F, MR-L, and FP-C contributed to the initial draft of the manuscript. All authors were involved in the critical revision of the manuscript and read and approved the final version of the manuscript for publication.

## Conflict of Interest

MO was employed by Biosearch Life SA. The remaining authors declare that the research was conducted in the absence of any commercial or financial relationships that could be construed as a potential conflict of interest.

## Publisher’s Note

All claims expressed in this article are solely those of the authors and do not necessarily represent those of their affiliated organizations, or those of the publisher, the editors and the reviewers. Any product that may be evaluated in this article, or claim that may be made by its manufacturer, is not guaranteed or endorsed by the publisher.
