# Supplementary material for: Staphylococcus epidermidis’ Overload During Suckling Impacts the Immune Development in Rats
Source: Front Nutr. 2022 Jul 4;9:916690. doi: 10.3389/fnut.2022.916690 (PMC9289531; doi:10.3389/fnut.2022.916690)
Supplement: Supplementary file 1 [file Data_Sheet_1.docx]

Supplementary Material

# Supplementary Material

**Supplementary Figure 1.** Body weight (g) of males (a), females (b), low weight (c) and high weight (d) rats during the study (from day 2 to day 42 of life). Results are expressed as mean ± SEM (n = 12 animals/group). REF: reference group; Ls: group supplemented with low dose of S. epidermidis and Hs: group supplemented with high dose of S. epidermidis.

**Supplementary Table 1.** Growth-associated measurements and relative weight of organs at the end of the supplementation (day 21 of life) and three weeks after (day 42 of life).

|  | **REF** | **Ls** | **Hs** |
| --- | --- | --- | --- |
| **Day 21** |  |  |  |
| Naso-anal length (body, cm) | 12.3 ± 0.1 | 12.2 ± 0.1 | 12.3 ± 0.2 |
| Anus-tail length (tail, cm) | 7.0 ± 0.1 | 6.9 ± 0.1 | 6.9 ± 0.2 |
| Naso-tail length (cm) | 19.2 ± 0.2 | 19.1 ± 0.2 | 19.2 ± 0.3 |
| Body/Tail length ratio | 1.8 ± 0.0 | 1.8 ± 0.0 | 1.8 ± 0.0 |
| Body mass index (g/cm^2^) | 0.4 ± 0.0 | 0.3 ± 0.0 | 0.4 ± 0.0 |
| Lee index (g^0.33^/cm, x1000) | 307.4 ± 2.5 | 304.7 ± 1.8 | 307.2 ± 2.3 |
| Spleen weight/BW (%) | 0.6 ± 0.0 | 0.6 ± 0.0 | 0.6 ± 0.0 |
| Thymus weight / BW (%) | 0.5 ± 0.0 | 0.5 ± 0.0 | 0.5 ± 0.0 |
| Kidney weight / BW (%) | 0.6 ± 0.0 | 0.6 ± 0.0 | 0.5 ± 0.0 |
| Liver weight / BW (%) | 4.3 ± 0.1 | 4.3 ± 0.1 | 4.4 ± 0.1 |
| Stomach weight / BW (%) | 0.9 ± 0.0 | 0.9 ± 0.0 | 0.9 ± 0.0 |
| Small int. weight / BW (%) | 4.5 ± 0.1 | 4.4 ± 0.1 | 4.4 ± 0.1 |
| Large int. weight / BW (%) | 2.1 ± 0.1 | 2.1 ± 0.1 | 2.0 ± 0.1 |
| Small int. length/ BW (cm/g) | 94.5 ± 3.1 | 100.2 ± 3.9 | 98.4 ± 5.2 |
| Large int. length/ BW (cm/g) | 21.2 ± 1.2 | 23.2 ± 1.5 | 23.8 ± 1.7 |
| **Day 42** |  |  |  |
| Naso-anal length (body, cm) | 18.8 ± 0.2 ^δ^ | 19.5 ± 0.3 ^* δ^ | 19.5 ± 0.2 ^* δ^ |
| Anus-tail length (tail, cm) | 14.0 ± 0.2 ^δ^ | 14.3 ± 0.2 ^δ^ | 14.2 ± 0.2 ^δ^ |
| Naso-tail length (cm) | 32.8 ± 0.4 ^δ^ | 33.8 ± 0.4 ^δ^ | 33.6 ± 0.3 ^δ^ |
| Body/Tail length ratio | 1.3 ± 0.0 ^δ^ | 1.4 ± 0.0 ^δ^ | 1.4 ± 0.0 ^δ^ |
| Body mass index (g/cm^2^) | 0.5 ± 0.0 ^δ^ | 0.5 ± 0.0 ^δ^ | 0.5 ± 0.0 ^δ^ |
| Lee index (g^0.33^/cm, x1000) | 295.5 ± 1.8 ^δ^ | 293.8 ± 1.4 ^δ^ | 290.5 ± 1.6 ^δ^ |
| Spleen/BW ratio (%) | 0.4 ± 0.0 ^δ^ | 0.4 ± 0.0 ^δ^ | 0.4 ± 0.0 ^δ^ |
| Thymus/ BW ratio (%) | 0.4 ± 0.0 ^δ^ | 0.3 ± 0.0 ^δ^ | 0.4 ± 0.0 ^δ^ |
| Kidneys/ BW ratio (%) | 0.5 ± 0.0 ^δ^ | 0.5 ± 0.0 ^δ^ | 0.5 ± 0.0 ^δ^ |
| Liver/ BW ratio (%) | 4.6 ± 0.1 ^δ^ | 4.7 ± 0.1 ^δ^ | 4.6 ± 0.1 ^δ^ |
| Stomach/ BW ratio (%) | 0.7 ± 0.0 ^δ^ | 0.7 ± 0.0 ^δ^ | 0.7 ± 0.0 ^δ^ |
| Small int./ BW ratio (%) | 4.3 ± 0.1 | 4.5 ± 0.1 | 4.5 ± 0.2 |
| Large int./ BW ratio (%) | 6.5 ± 0.4 ^δ^ | 6.4 ± 0.2 ^δ^ | 6.0 ± 0.2 ^δ^ |
| Small int. length/ BW (cm/g) | 34.1 ± 1.4 ^δ^ | 33.8 ± 0.8 ^δ^ | 32.7 ± 1.0 ^δ^ |
| Large int. length/ BW (cm/g) | 10.2 ± 0.4 ^δ^ | 10.0 ± 0.3 ^δ^ | 9.9 ± 0.4 ^δ^ |

Relative weight of organs is expressed as percentage (%) with respect to the body weight (BW) and growth-associated measurements are expressed as mean ± SEM (n = 12). Statistical significance: * p<0.05 *vs*. REF, ^δ^day 42 *vs*. day 21. REF: the reference group; Ls: group supplemented with low dose of *S. epidermidis* and Hs: group supplemented with high dose of *S. epidermidis*.

**Supplementary Table 2.** Percentage of immunoglobulins in plasma at the end of the supplementation (day 21 of life) and three weeks later (day 42 of life; the end of the study).

| **Ig type (%)** | **REF** | **Ls** | **Hs** |
| --- | --- | --- | --- |
| **Day 21** |  |  |  |
| IgG | 96.7 ± 0.3 | 95.7 ± 0.3 ^*^ | 97.0 ± 0.2 ^#^ |
| IgG1 | 3.8 ± 0.3 | 4.7 ± 0.4 | 7.1 ± 0.8 ^*#^ |
| IgG2a | 17.6 ± 1.7 | 21.8 ± 1.6 | 20.6 ± 0.8 |
| IgG2b | 48.7 ± 1.7 | 38.0 ± 2.5 ^*^ | 35.8 ± 2.2 ^*^ |
| IgG2c | 26.7 ± 3.3 | 31.2 ± 1.8 | 33.5 ± 2.4 |
| IgM | 2.6 ± 0.3 | 3.7 ± 0.3 ^*^ | 2.4 ± 0.2 ^#^ |
| IgA | 0.7 ± 0.1 | 0.8 ± 0.1 | 0.7 ± 0.1 |
| **Day 42** |  |  |  |
| IgG | 95.6 ± 0.3 ^δ^ | 95.3 ± 0.3 ^δ^ | 95.5 ± 0.3 ^δ^ |
| IgG1 | 2.6 ± 0.2 ^δ^ | 2.8 ± 0.3 ^δ^ | 3.4 ± 0.4 ^δ^ |
| IgG2a | 5.8 ± 0.5 | 8.2 ± 0.9 ^* δ^ | 8.3 ± 0.9 ^* δ^ |
| IgG2b | 16.2 ± 1.2 ^δ^ | 18.7 ± 1.9 ^δ^ | 18.2 ± 1.8 ^δ^ |
| IgG2c | 70.9 ± 1.5 ^δ^ | 65.6 ± 2.7 ^δ^ | 65.7 ± 2.3 ^δ^ |
| IgM | 3.8 ± 0.3 ^δ^ | 4.1 ± 0.3 ^δ^ | 3.9 ± 0.3 ^δ^ |
| IgA | 0.7 ± 0.1 | 0.6 ± 0.1 | 0.6 ± 0.1 |

The percentage of 100% of the isotypes corresponds to the sum of IgG+IgM+IgA and the percentage 100% of the subtypes of IgG corresponds to the sum of IgG1+IgG2a+IgG2b+IgG2c. Results are expressed as mean ± SEM (n = 12). Statistical significance: ^*^p<0.05 *vs*. REF, ^#^ Hs *vs.* Ls. REF: reference group; Ls: group supplemented with low dose of *S. epidermidis* and Hs: group supplemented with high dose of *S. epidermidis*.
